# Structural Transformations of Amino-Acid-Based Polymers: Syntheses and Structural Characterization

**DOI:** 10.3390/polym10040360

**Published:** 2018-03-23

**Authors:** Tien-Wen Tseng, Tzuoo-Tsair Luo, Hsiao-Shan Chiu, Chih-Chieh Wang, Gene-Hsiang Lee, Hwo-Shuenn Sheu, Kuang-Lieh Lu

**Affiliations:** 1 Department of Chemical Engineering and Biotechnology, National Taipei University of Technology, Taipei 106, Taiwan; luott@ntpc.edu.tw; 2 Institute of Chemistry, Academia Sinica, Taipei 115, Taiwan; anhhhh33@gmail.com; 3 Department of Chemistry, Soochow University, Taipei 100, Taiwan; 4 Department of Chemistry, National Taiwan University, Taipei 106, Taiwan; ghlee@ntu.edu.tw; 5 National Synchrotron Radiation Research Center, Hsinchu 300, Taiwan; hsheu@nsrrc.org.tw

**Keywords:** amino-acid-based coordination polymers, structural transformations, homochiral helices, self-assembly process, l-thioproline

## Abstract

A discrete complex [Zn(tpro)_2_(H_2_O)_2_] (**1**, Htpro = l-thioproline), and two structural isomers of coordination polymers, a 1D chain of [Zn(tpro)_2_]_n_ (**2**) and a layered structure [Zn(tpro)_2_]_n_ (**3**), were synthesized and characterized. The discrete complex **1** undergoes a temperature-driven structural transformation, leading to the formation of a 1D helical coordination polymer **2**. Compound **3** is comprised of a 2D homochiral layer network with a (4,4) topology. These layers are mutually linked through hydrogen bonding interactions, resulting in the formation of a 3D network. When **1** is heated, it undergoes nearly complete conversion to the microcrystalline form, i.e., compound **2**, which was confirmed by powder X-ray diffractions (PXRD). The carboxylate motifs could be activated after removing the coordinated water molecules by heating at temperatures of up to 150 °C, their orientations becoming distorted, after which, they attacked the activation sites of the Zn(II) centers, leading to the formation of a 1D helix. Moreover, a portion of the PXRD pattern of **1** was converted into the patterns corresponding to **2** and **3**, and the ratio between **2** and **3** was precisely determined by the simulation study of in-situ synchrotron PXRD expriments. Consequently, such a 0D complex is capable of underdoing structural transformations and can be converted into 1D and/or 2D amino acid-based coordination polymers.

## 1. Introduction

Various strategies for the rational design of coordination polymers (CPs) or metal-organic frameworks (MOFs) have been subjects of considerable interest recently, not only because of their structural diversities and intriguing topologies [1,2,3,4,5,6], but also due to potential applications in the fields of gas storage, separation, fluorescence, drug delivery, and related fields [7,8,9,10,11,12]. The creation of desirable CPs or MOFs with variable dimensions relies on controlling a few critical factors, such as the functionality on the ligand, concentration, template, pH, temperature, the solvent system being used, and reaction time, among others [13,14,15,16,17]. One unusual feature of CPs or MOFs is their structural flexibilities, which can lead to remarkable effects, including breathing, pore-discriminating adsorption and structural transformations in the solid state, which are completely different from those that are observed in solvent systems [18,19,20,21,22,23]. Of particular interest are structural transformations that are involved with the replacement or the substitution of labile ligands, which provide crystallographic snapshots that are potentially useful for collecting information related to solvent-free chemical reactions [24,25,26,27]. The coordination geometries of the metal ions could be changed upon exposure to exogenous stimuli, and some coordinating tectons can escape via metal-ligand bonding breakage, thus facilitating the reforming of new bonding modes in a concerted manner [28,29,30]. Recent achievements in phase transformations include demonstrations of the mutual sliding of coordination arrays in the solid state, which may be induced by external stimuli, such as solvents, ions, light, temperature, and mechanical force, and synergistic effects associated with these stimuli [31,32,33,34,35,36,37,38]. For example, the reversible solvent-responsive phase transformation process driven by water molecules was explored for the case of a multi-metallic coordination polymer [39]. For some time, we have been interested in preparing labile compounds containing a chiral amino acid ligand as target metal complexes, which can be self-assembled under mild conditions. There are several structural transformations that are primarily influenced by thermal association, condensation, and the rearrangement of bonds [40,41,42,43,44,45]; however, employing such amino acid-containing complexes in studies of their self-reorganizing properties as well as their structural transformations have not fully been addressed [46,47,48,49]. A detailed understanding of the behaviors of such complexes is thus important in the design of future materials for use in advanced applications.

As part of our ongoing efforts in the design and synthesis of functionally crystalline materials [49,50,51,52,53], we report herein on the preparation and characterization of one discrete [Zn(tpro)_2_(H_2_O)_2_] complex (**1**, Htpro = l-thioproline), and two structural isomers of coordination polymers, one comprised of a 1D chain of [Zn(tpro)_2_]_n_ (**2**) and the other with a layered structure [Zn(tpro)_2_]_n_ (**3**). Their features are as follows: (i) the discrete complex contains two labile water molecules and two strongly chelated amino acid ligands; (ii) compounds **2** and **3** have the same fomula, but their structures and space groups are completely different, the former is a 1D right-handed helix, while the latter is composed of a 2D protuberant-grid-type network; (iii) the use of several different solvents or the addition of structure-directing reagents was found to play an important role in promoting the yield of each product; (iv) upon heating at 210 °C, the carboxylate oxygens of the complexes became distorted and coordinated to the neighboring Zn(II) centers, i.e., the activation sites, leading to the PXRD pattern of **1** being transformed to that of **2**; and (v) a small fraction of **3** (2.4%) could also be obtained, as verified by the studies of in situ synchrotron powder X-ray diffraction patterns. Furthermore, this methodology provides an opportunity to develop an effective strategy for preparing a variety of diverse products under mild conditions. This report deals with the issue of labile ligands that can be removed upon heating. Importantly, such a discrete complex exhibited structural transformations via conversion to 1D and/or 2D amino acid-based coordination polymers in the solid state.

## 2. Experimental

### 2.1. Materials and Physical Techniques

All chemicals were of reagent grade and used as commercially obtained without further purification. Elemental analyses (carbon, hydrogen and nitrogen) were performed using a Perkin-Elmer 2400 elemental analyzer (Perkin-Elmer, Inc., Billerica, MA, USA). The infrared spectrum was recorded on a Nicolet Fourier Transform IR (Nicolet Instrument Inc., Madison, WI, USA), and a Nicolet MAGNA-IR 500 spectrometer (Nicolet Instrument Inc., Madison, WI, USA) in the range 500–4000 cm^−1^ using the KBr disc technique. Thermogravimetric analyses (TGA) were performed on a computer-controlled Perkin-Elmer 7 Series/UNIX TGA7 analyzer (Perkin-Elmer, Inc., Billerica, MA, USA). Single-phased powder samples of **1** (3.1 mg), **2** (3.2 mg) and **3** (3.4 mg) were loaded into alumina pans and heated with a ramp rate of 5 °C/min from room temperature to 800 °C under nitrogen atmosphere.

### 2.2. Synthesis of [Zn(Tpro)_2_(H_2_O)_2_] *(**1**)*

An ethanol solution (4 mL) of Zn(CH_3_COO)_2_·2H_2_O (0.100 mmol, 0.0223 g) was carefully layered on the top of an aqueous solution (4 mL) of triethylamine (0.6 mL, 0.3 mmol) and l-thioproline (Htpro, 0.359 mmol, 0.0488 g). The resulting solution was then allowed to stand under ambient conditions for several days, whereupon colorless plate-shaped crystals of **1** were formed in 93% yield (0.034 g, based on Zn(II)). Elemental analysis calcd (%) for C_8_H_16_N_2_O_6_S_2_Zn (**1**): C 26.13, H 7.68, N 3.86. Found: C 26.27, H 7.65, N 4.40. IR (KBr pellet): *ν* = 1325 (s), 1402 (s), 1477 (s), 1599 (vs), 1689 (s), 2938 (s), 3204 (s) cm^−1^. It is noteworthy that complex **1** was obtained as the major product, and the two other products were formed in minor amounts under mild conditions except that mixing was necessary, suggesting that the laying methodology may play an important role in the synthesis of complex **1**.

### 2.3. Synthesis of [Zn(tpro)_2_]_n_ *(**2**)*

An ethanol solution (4 mL) of Zn(CH_3_COO)_2_·2H_2_O (0.100 mmol, 0.0223 g) was carefully layered on the top of a solution, consisting of an aqueous solution (4 mL) of Htpro (0.359 mmol, 0.0488 g) and triethylamine (0.6 mL, 0.3 mmol), and an ethanol solution (4 mL) of 4,4′-bipyridine (0.0497 g, 0.312 mmol). The resulting solution was then allowed to stand at ambient conditions for two weeks, whereupon colorless cubic block crystals were formed in 79% yield (0.026 g, based on Zn(II)). Elemental analysis calcd (%) for C_8_H_12_N_2_O_4_S_2_Zn (**2**): C 29.12, H 8.50, N 3.64. Found: C 29.18, H 8.49, N 3.65. IR (KBr pellet): *ν* = 1336 (m), 1370 (m), 1608 (vs), 1645 (s), 3218 (s), 3444(s) cm^−1^.

### 2.4. Synthesis of [Zn(tpro)_2_]_n_ *(**3**)*

A methanol solution (4 mL) of Zn(CH_3_COO)_2_·2H_2_O (0.100 mmol, 0.0223 g) was carefully layered on the top of an aqueous solution (4 mL) of Htpro (0.359 mmol, 0.0488 g) and triethylamine (0.6 mL, 0.3 mmol) and a methanol solution (4 mL) of 4,4′-bipyridine (0.0469 g, 0.294 mmol). The resulting solution was then allowed to stand at ambient conditions for several days, colorless grainy crystals of **3** were obtained in 88% yield (0.029 g, based on Zn(II)). Elemental analysis calcd (%) for C_8_H_12_N_2_O_4_S_2_Zn (**3**): C 29.12, H 8.50, N 3.64. Found: C 29.14, H 8.47, N 3.65. IR (KBr pellet): *ν* = 1326 (s), 1403 (s), 1431 (s), 1476 (s), 1598 (vs), 1692 (s), 2939 (s), 3206 (s) cm^−1^.

### 2.5. Crystallographic Data Collection and Refinement

Single crystal X-ray diffraction analyses of compounds **1**–**3** were performed on a Siemens SMART diffractomer (Siemens, Germany) with a CCD detector with Mo K_α_ radiation (λ = 0.71073 Å) at 295(2) K. Cell parameters were retrieved using SMART [54] software (Bruker SAINT, Tokyo, Japan) and refined with SAINT on all observed reflections. Data reduction was performed with the SAINT software and corrected for Lorentz and polarization effects. Absorption corrections were applied with the program SADABS (Bruker (2016), Tokyo, Japan). Direct phase determination and subsequent difference Fourier map synthesis yielded the positions of all non-hydrogen atoms, which were subjected to anisotropic refinements. All hydrogen atoms were generated geometrically with the exception of the hydrogen atoms of the coordinated water molecules, which were located in the difference Fourier map with the corresponding positions and isotropic displacement parameters being refined. The final full-matrix, least-squares refinement on *F*^2^ was applied for all observed reflections [*I* > 2σ(*I*)]. All calculations were performed using the SHELX software package (SHELXS-97, Göttingen, Germany) [55]. The Crystal data and structural refinement for compounds **1**–**3** are listed in Table 1. Selected bond and hydrogen bonding lengths (Å) and angles (°) for **1**–**3** are summarized in Tables S1–S6. The Cambridge Crystallographic Data Centre CCDC-1823036, 1823037 and 1823039 for **1**, **2** and **3**, respectively, contain the supplementary crystallographic data for the paper. These data can be obtained free of charge at www.ccdc.cam.ac.uk/conts/retrieving.html or from the Cambridge Crystallographic Data Centre (Cambridge, UK).

### 2.6. Powder X-ray Diffraction of Compounds ***1**–**3***

The powder X-ray diffraction patterns of **1** were recorded by the National Synchrotron Radiation Research Center (NSRRC, TPS 09A, Hsinchu, Taiwan) via the BL01C2 beamline. The ring energy of NSRRC was operated at 1.5 GeV with a typical current of 300 mA. The wavelength of the incident X-rays was 1.0332 Å (12.0 keV), delivered from the superconducting wavelength-shifting magnet and a Si(111) double-crystal monochromator. The diffraction patterns were recorded with a Mar345 imaging plate detector approximately 300 mm from sample positions and typical exposure duration 5 min. The pixel size of Mar345 was 100 µm. The one-dimensional powder diffraction profile was converted with program FIT2D (ESRF, Grenoble, France) and cake-type integration. The diffraction angles were calibrated according to Bragg positions of Ag-Benhenate and Si powder (NBS640b) standards. In situ synchrotron powder X-ray diffraction patterns of **1**–**3** were recorded from room temperature to 450 °C with a heating rate approximately 10 °C/min. The powder sample was sealed in a capillary (1.0 mm diameter) and heated in a stream of hot air; each in situ powder XRD pattern was exposed for about 1.2 min.

## 3. Results and Discussion

### 3.1. Syntheses of Compounds ***1**–**3***

**Scheme 1:**
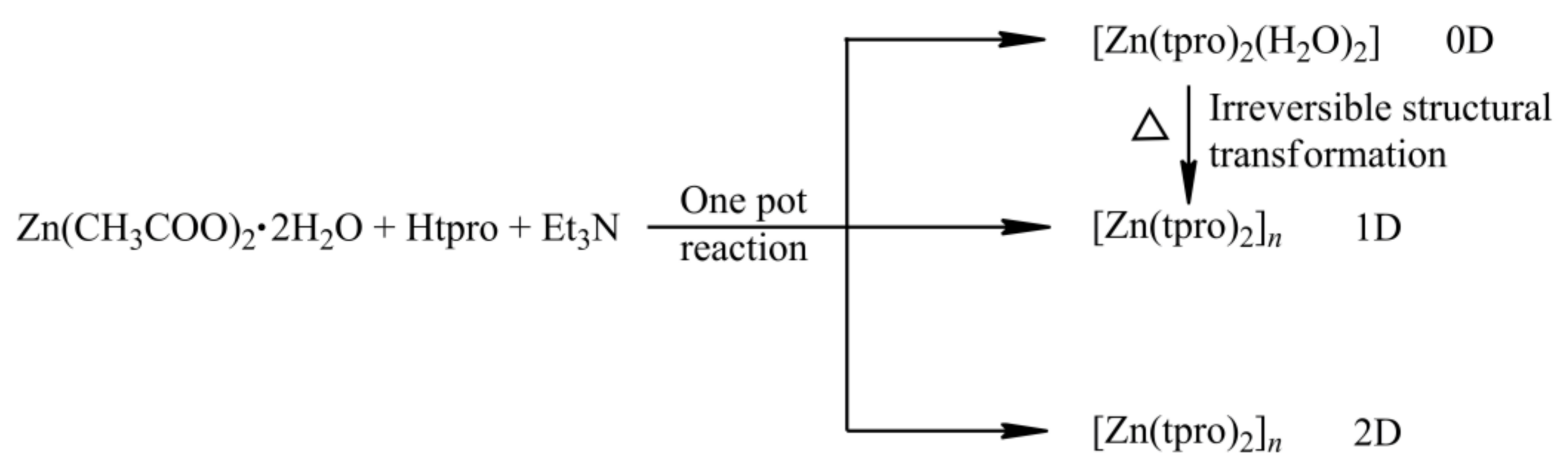
The syntheses of Compounds **1**–**3.**

**Figure 1:**
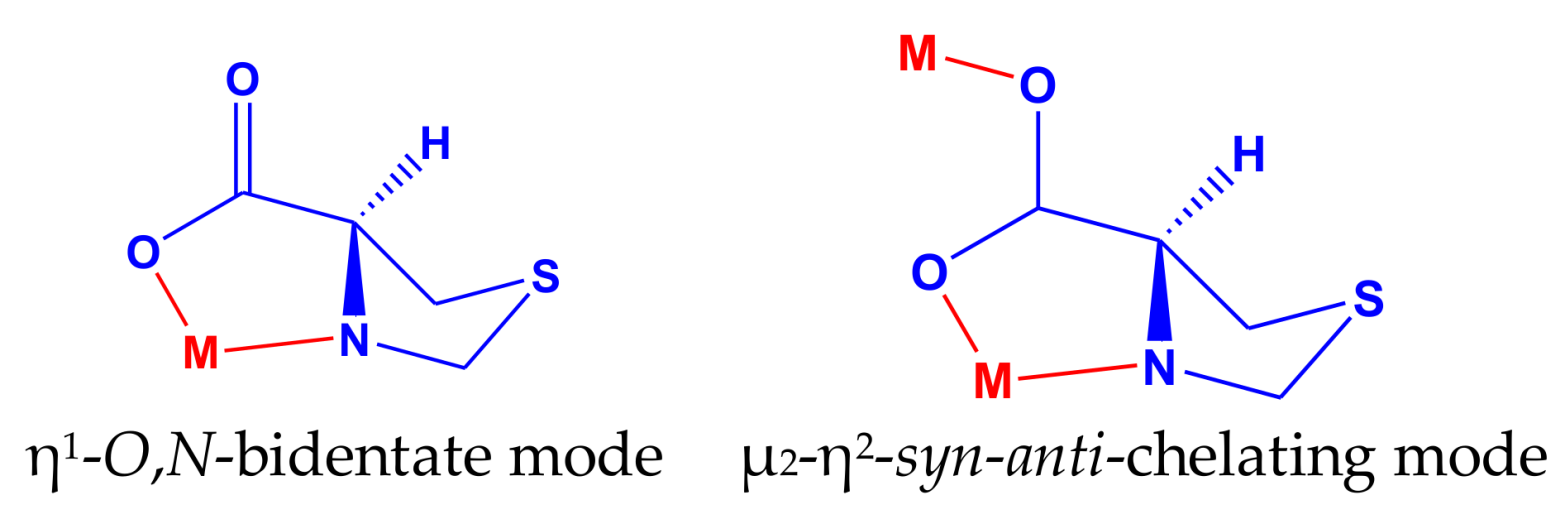
Available coordination modes between the tpro^−^ Ligand and the metal ion (M^n+^).

A discrete complex [Zn(tpro)_2_(H_2_O)_2_] (**1**, Htpro = l-thioproline), and two structural isomers of coordination polymers, a 1D chain of [Zn(tpro)_2_]_n_ (**2**) and a layered structure [Zn(tpro)_2_]_n_ (**3**), were synthesized by reacting Zn(CH_3_COO)_2_·2H_2_O, l-thioproline (Htpro) and triethylamine at ambient temperature for several days through a single-step, self-organization process (Scheme 1). Interestingly, three different single crystal products could be obtained under similar conditions by appropriately tuning the system via the use of different solvents, such as ethanol, methanol, aqueous solutions, and introducing a structure-directing reagent, such as 4,4′-bipyridine. In order to introduce the chirality into the products, we attempted to use a l-thioproline species as a ligand to achieve self-assembly with Zn(II) ions under mild conditions. Because the five-membered ring of the thiazolidine motif of Htpro is not planar, it exists in a twisted type of conformation. The tpro^−^ ligand can adopt two types of bonding modes: a η^1^-*O*,*N*-bidentate mode or a µ_2_-η^2^-*syn-anti*-chelating mode (Figure 1).

It should be noted that the tpro^−^ ligand can exist in a chelating mode, thus allowing it to be linked in La helical manner, permitting its chirality to be translated into the target products. The thio and amino functional groups of the tpro^−^ ligand can also participate in hydrogen bonding interactions, thus increasing the dimensions of the target products. However, such tpro-based complexes are still rare as of this writing [56,57,58,59]. A chiral l-thioproline species was employed as a ligand to react with the Zn(II) ions under mild conditions, in order to permit some labile compounds with coordinated water molecules to be produced. Thus, such a simple labile complex could be used to investigate the intriguing structural transformations.

### 3.2. Structural Description of [Zn(Tpro)_2_(H_2_O)_2_] *(**1**)*

**Figure 2:**
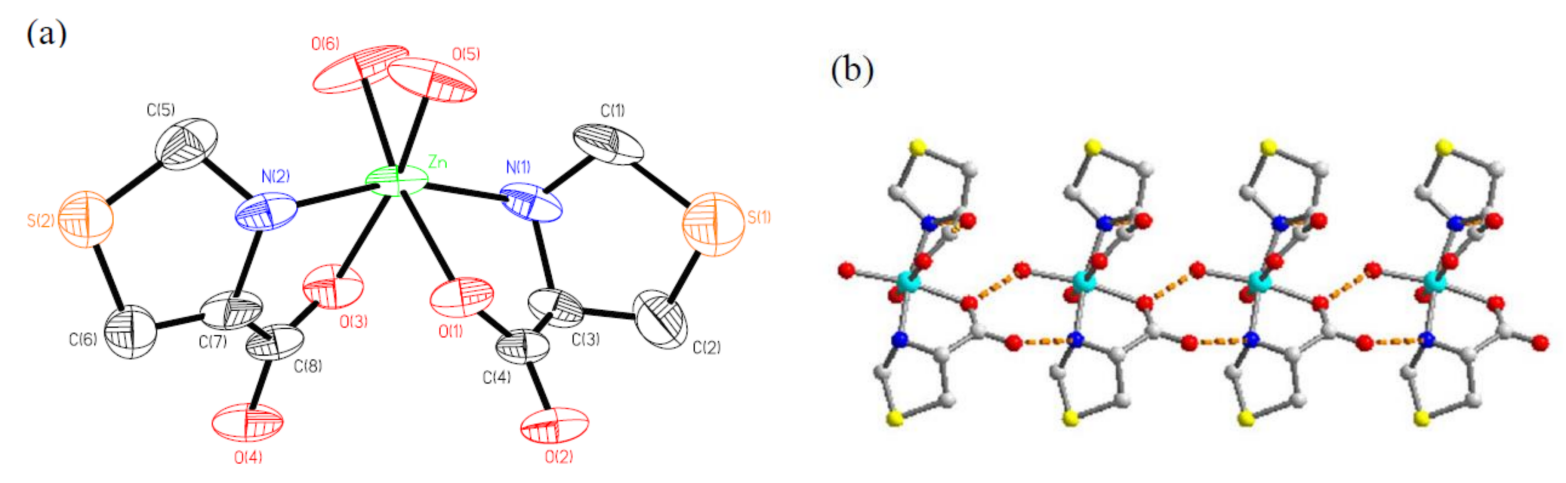
Structures of **1**: (**a**) coordination environment of the Zn(II) ion; (**b**) a simplified 1D chain connected by hydrogen bonding interactions (orange dash lines).

A single-crystal X-ray diffraction analysis revealed that compound **1** crystallizes in the monoclinic space group *P*2_1_. As shown in Figure 2a, the Zn(II) ion is bound to two nitrogen atoms (N1,N2) and two oxygen atoms (O1,O3) from two crystallographically independent tpro^−^ ligands, and two oxygen atoms (O5, O6) from two coordinated water molecules, giving rise to a complex with a distorted octahedral geometry. Two coordinated water and two tpro^−^ ligands are located in a cis conformation, and the latter adopted a chelating coordination mode. Compound **1** can be classified as a class of MA_2_B_2_C_2_ type complex, thus, from a stereochemical point of view, it could be described as having a *trans*-*N*,*N*, *cis*-*O*,*O*, *cis*-*O*′,*O*′ configuration. The Zn–O distances are in the range of 2.051(7)–2.158(6) Å and the Zn–N distances are 2.146(1) and 2.143(1) Å, respectively. It is noteworthy that a variety of hydrogen bonding interactions are operative among these discrete complexes. They were mutually interlinked via the coordinated water molecules (O5, O6) and the carboxylate oxygen atoms (O3, O2, O4) from the tpro^−^ ligands with hydrogen bonding interactions (O–H···O) with distances of 2.64, 2.73 and 2.69 Å, respectively. As a result, these Zn^2+^ ions are linked together through the carboxylate motif of the tpro^−^ ligands, leading to the formation of a 1D chain (Figure 2b), and these chains were then further staggered in an ABAB manner, resulting in the formation of a 2D layer (Figure S1). Finally, these hydrogen-bonded layers were further linked by hydrogen bonding interactions (C–H···S) with distances of 3.764 (1) and 3.948 (2) Å, resulting in a 3D supramolecular architecture, as depicted in Figure S2.

### 3.3. Structural Description of [Zn(tpro)_2_]_n_ *(**2**)*

**Figure 3:**
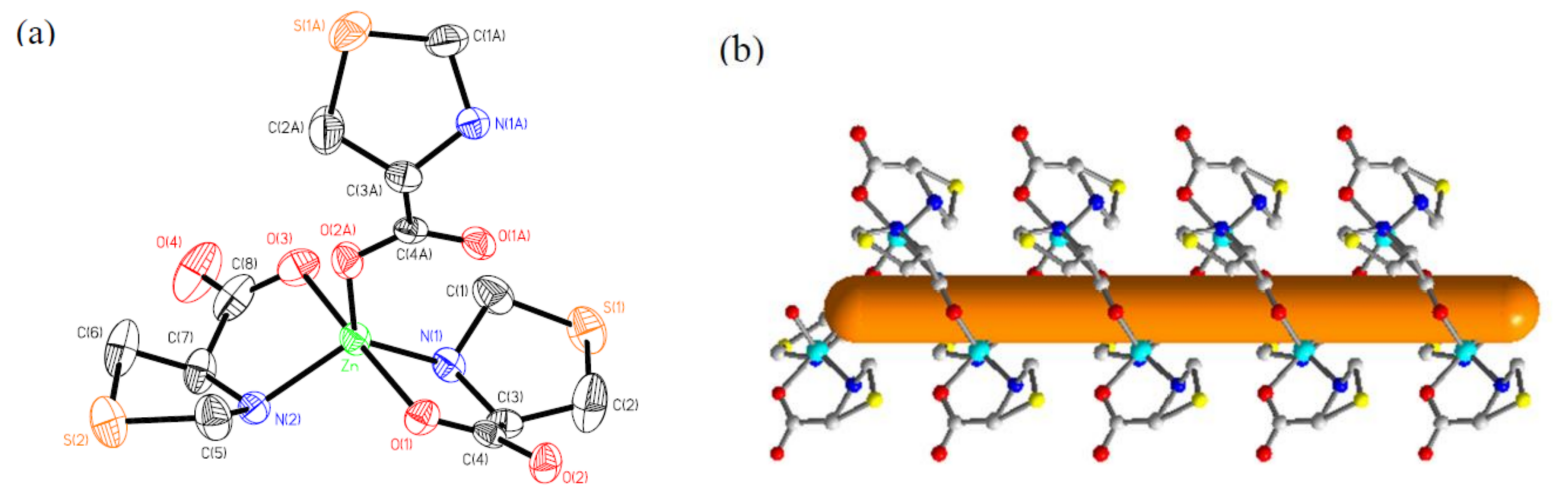
Structures of compound **2**: (**a**) the coordination environment of the Zn(II) ion; (**b**) a simplified right-handed single-stranded helical chain.

A single-crystal X-ray diffraction analysis revealed that compound **2** also crystallizes in the monoclinic space group *P*2_1_, but the unit cell parameters of this compound were completely different from those of **1**. As shown in Figure 3, the Zn(II) center is surrounded by three tpro^−^ ligands using the N atom and an O atom in a µ_2_-η^2^-*syn*-*anti*-chelating mode to produce slightly distorted trigonal bipyramidal coordination geometry, in which the O1 and O3 atoms occupy axial positions and the other atoms O2, N1 and N2 are located in equatorial positions. The Zn–O distances are 2.004(3), 2.053(3) and 2.128(3) Å and the Zn–N distances are 2.055(3) and 2.080(3) Å. Notably, in compound **2**, the O2 atom from the carboxylate motif of the tpro^−^ ligand served as a bridge for the neighboring Zn(II) ions, resulting in the formation of a 1D right-handed helix (Figure 3b). These adjacent helical chains were further connected via hydrogen bonding interactions (N–H⋅⋅⋅O) with distances of 2.862(5), 3.170(5) and 3.097(6) Å, forming a 2D homochiral layer structure. Finally, these layers were linked together by hydrogen bonding through C–H⋅⋅⋅S interactions, leading to the formation of a 3D supramolecular structure (Figure S3).

### 3.4. Structural Description of [Zn(tpro)_2_]_n_ *(**3**)*

**Figure 4:**
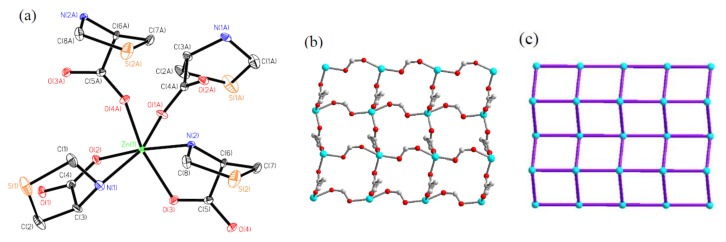
Structures of compound **3**: (**a**) the coordination environment of Zn(II) ion; (**b**) a simplified 2D homochiral layer with the Zn(II) ions and the carboxylate motifs from the tpro^−^ ligands in the *ab* plane, while the other motifs are omitted for clarity; (**c**) a 2D layer structure with a (4,4) topology.

A single-crystal X-ray diffraction analysis revealed that compound **3** crystallizes in the orthorhombic space group *P*2_1_2_1_2_1_. As shown in Figure 4a, the Zn(II) ion is six-coordinated via two N and two O atoms of two chelated tpro^−^ ligands, and two O atoms from two η^1^-carboxylate from two tpro^−^ ligands, in a distorted octahedral geometry. It is interesting to note that there are four tpro^−^ ligands bound to the Zn(II) ions, two ligands are coordinated via N and O atoms adopting a bidentate mode, and thereby forming two five-membered chelated rings. The other two are coordinated through two η^1^-carboxylate motifs in a η**^1^**-*O*,*N*-bidentate mode, each of which is located trans to the bidentate tpro^−^ ligand. The Zn–O distances are 2.030(2), 2.081(2), 2.099(2) and 2.131(2) Å, respectively, and the Zn–N distances are 2.149(2) and 2.289(2) Å. Interestingly, all of the tpro^−^ ligands are linked to two Zn(II) centers in a µ_2_-η^2^-*syn*-*anti*-chelating mode, forming a right-handed helical structure. The two types of helices (along the *a*, *b* axis) intersect through the Zn(II) centers, yielding a 2D homochiral layer in the *ab* plane. The Zn^II^ atom can be regarded as a four-connecting node. The carboxylate motifs of the tpro^−^ ligands function as an extended linker, bridging two Zn^II^ centers, while the other motifs are omitted for the sake of clarity. Interestingly, a protuberant-grid-type network is apparent (Figure 4b). Thus, this layered architecture possesses a (4,4) topology (Figure 4c). The Zn^II^···Zn^II^ separation distances across the µ_2_-η^2^-*syn*-*anti*-COO^−^ are 5.883(1) and 5.226(1) Å, respectively. These layers are stacked in an ABAB manner connected by hydrogen bonding interactions (N–H···S) with a distance of 3.418(2) Å, leading to the formation of a 3D homochiral supramolecular network (Figure S4).

### 3.5. The Study of Thermogravimetric Analysis and Powder X-ray Diffraction

Thermogravimetric analyses (TGA) of **1** indicated that the coordinated water molecules are removed in the temperature range of 66−106 °C (Figure S5a). The resulting 9.8% weight loss is consistent with the calculated value (9.0%). After the water molecules are removed, the complex was stable up to a temperature of about 213 °C. TGA patterns of **2** showed no significant change and it was thermally stable at temperatures of up to 209.6 °C, after which it gradually decomposed (Figure S5b). The TGA pattern of **3** revealed that it was thermally stable at temperatures of up to 215 °C, after which it underwent decomposition (Figure S5c).

**Figure 5:**
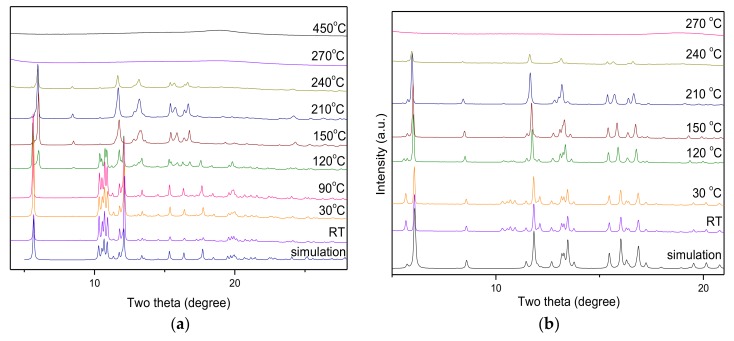
The PXRD patterns at different temperatures for compounds: **1** (**a**), **2** (**b**).

Powder X-ray diffraction (PXRD) patterns showed that the crystallinity of **1** does not change appreciably upon the removal of the coordinated water molecules at temperatures of up to 120 °C (Figure 5a). A new crystalline pattern, however, was observed when the temperature reached 150 °C, and this pattern remained unchanged at temperatures of up to 240 °C. However, at temperatures higher than 240 °C, a second new crystalline pattern appeared, indicating that some interesting structural changes had likely occurred. It is noteworthy that, when compound **1** was heated at 240 °C, the broad adsorption band of the O–H group (about at 3200 cm^−1^, in Figure S6a) changed, and a new spectrum appeared, which was similar to that of **2** (Figure S6b), indicating that **1** was converted into **2**. In addition, powder X-ray diffraction (PXRD) patterns showed that the crystallinity of **2** did not change appreciably until the temperature reached 240 °C, suggesting that this 1D polymeric chain is thermally stable at this temperature (Figure 5b). PXRD patterns of **3** showed that the crystallinity of **3** remains unchanged until the temperature reached 210 °C, indicating that it is relatively thermally stable (Figure S7).

### 3.6. Solid-State Thermal-Driven Structural Transformations

**Figure 6:**
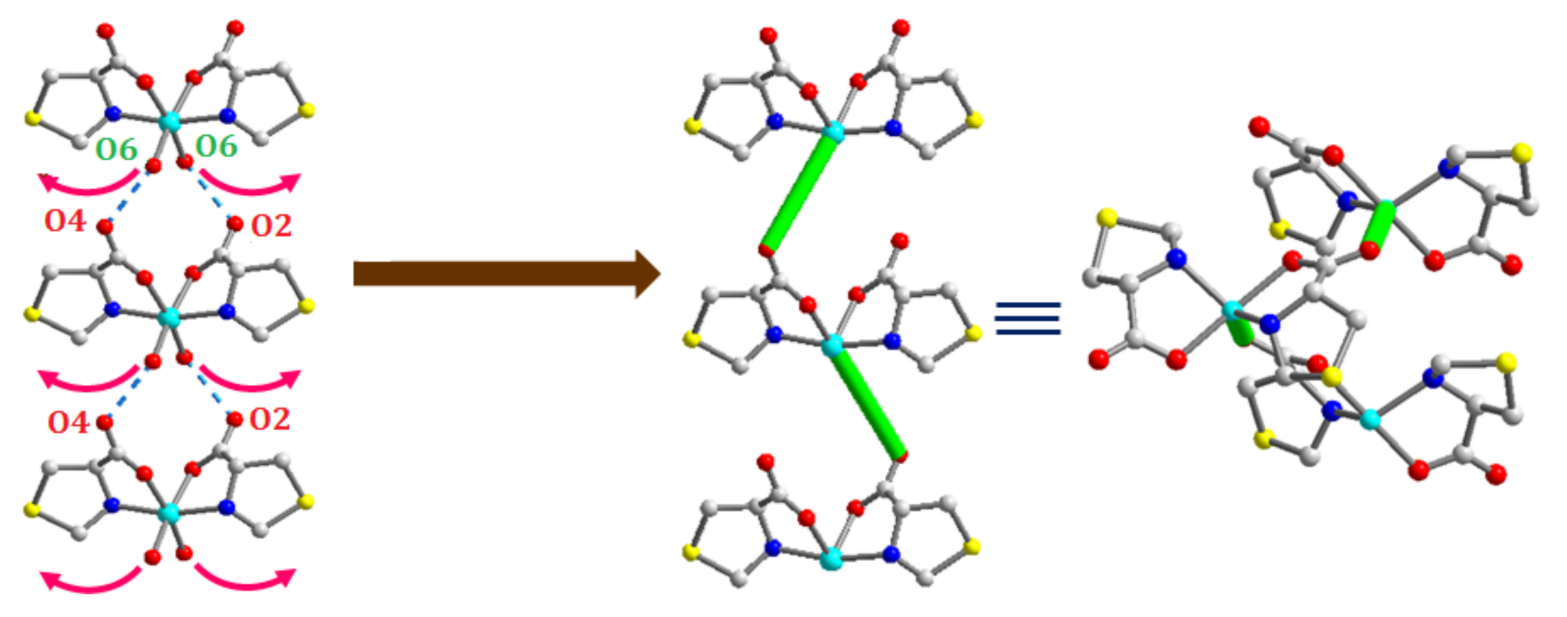
A plausible mechanism for compound **1** underwent a structural transformation. Upon the removal of labile water (O6), creating the activation sites on the Zn(II) centers and the neighbouring carboxylate oxygen atoms (O2, O4) form the tpro^−^ ligands were also activated and distorted to a suitable orientation, so that they were facilely attacked to the Zn(II) centers in the solid state.

We were astonished at what occurred during this thermally driven structural transformation in the solid state. We are inspired by the bottom-up approach that employs the assembly of secondary building units (SBUs), which play an important role in controlling the growth of higher dimensional metal-organic frameworks or coordination polymeric networks. It is noteworthy that, upon heating at 240 °C, the PXRD pattern of the dehydrated form of **1** underwent a complete change, suggesting that it was truly activated and that a structural transformation had occurred (Figure S8). As a result, upon the removal of the two coordinated water molecules, the Zn(II) centers of the complexes were transformed into two uncoordinated sites. These adjacent carboxylate oxygen atoms (O2, O4) from the neighboring complexes were probably slightly distorted and, as a result, were capable of being coordinated alternately to the active sites of Zn(II) ions, resulting in the structural transformation. (Figure 6, Figure S9 and S10).

**Figure 7:**
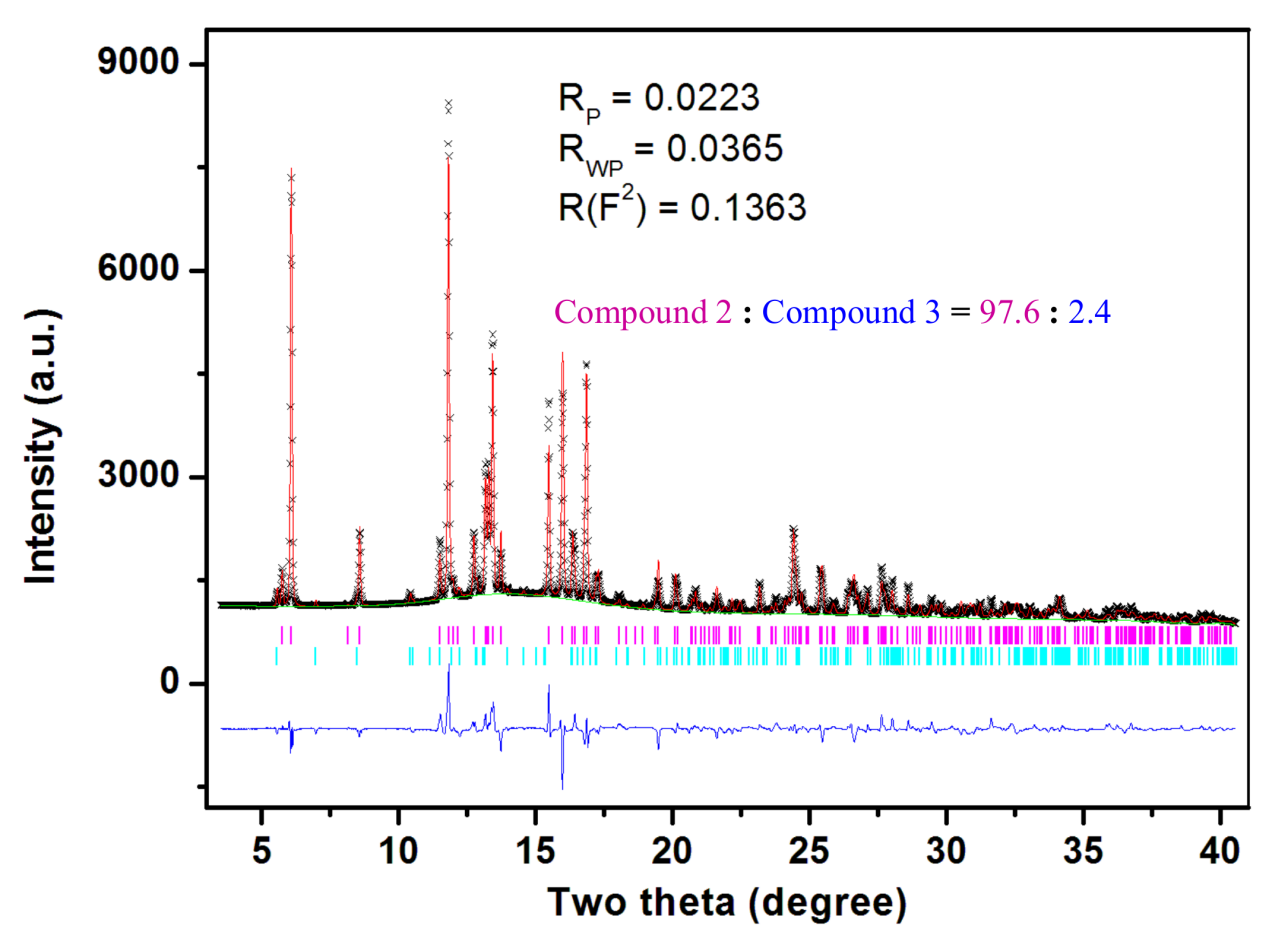
Representation of in situ synchrotron powder X-ray diffraction patterns of **1** during the phase transformations, indicating that compounds **2** and **3** were obtained in a ratio of 97.6% to 2.4%.

Finally, in order to further study the details involved in these phase transformations, we carried out a precise examination of **1** using in situ synchrotron powder X-ray diffraction. As shown in Figure 7, the simulation results of the synchrotron powder X-ray diffraction revealed that the ratio of **2**:**3** is 97.6% to 2.4%, indicating that small amounts of compound **3** were also produced during this structural transformation. This can be attributed to the fact that the orientations of carboxylate motifs of **2** were arranged in a helical form; as a result, it would be difficult for their related orientations to change in the solid state (Figure S11). Therefore, these in situ synchrotron powder X-ray diffraction analyses demonstrate that two phase transformations are induced from a discrete complex in the crystalline state. It is likely that, because of the close molecular packing, the restricted migration and reorientation of functional groups are able to form the higher-dimensional structures, but only sluggishly [60].

## 4. Conclusions

In conclusion, three compounds were successfully synthesized, including a discrete complex, a 1D right-handed helical chain and a 2D amino-acid-based coordination polymer comprised of a protuberant-grid-type network with a (4,4) topology. The labile water molecules of the discrete complex are removed upon heating, and the carboxylate oxygens become activated with their orientations being distorted. Consequently, it is possible to induce these carboxylate motifs from the neighbouring complexes to attack the Zn(II) ions with the activation sites. Therefore, the ligand, tpro^−^, which possesses chirality, and a carboxylate motif with a µ_2_-η^2^-*syn*-*anti*-chelating mode, appear to play an important role in the distortion effect that, in turn, facilitates structural transformations of the discrete complex into a 1D polymeric chain and a small amount of 2D structure. This study provides the deep insights into the structural transformation of such a discrete complex, which can be converted to 1D and/or 2D amino-acid-based coordination polymers.

## Figures and Tables

**Table 1:** Crystal data and refinement details of **1**–**3.**

Compounds	1	2	3
formula	C_8_H_16_N_2_O_6_S_2_Zn	C_8_H_12_N_2_O_4_S_2_Zn	C_8_H_12_N_2_O_4_S_2_Zn
*M*_w_	365.72	329.69	329.69
crystal system	monoclinic	monoclinic	orthorhombic
space group	*P*2_1_	*P*2_1_	*P*2_1_2_1_2_1_
*a* (Å)	6.0254(2)	9.714(1)	5.8839(5)
*b* (Å)	20.8382(9)	5.7126(6)	9.2587(8)
*c* (Å)	6.1614(2)	10.336(1)	21.211(2)
α (deg)	90.00	90.00	90.00
β (deg)	111.282(2)	93.187(2)	90.00
γ (deg)	90.00	90.00	90.00
*V* (Å^3^)	720.86(5)	572.7(1)	1157.5(2)
*Z*	2	2	4
*ρ*_calc_/g·cm^−3^	1.685	1.912	1.892
2θ range (deg)	3.91° to 27.50°	1.97° to 27.49°	1.92° to 27.50°
µ (mm^−1^)	2.014	2.512	2.486
*T*/K	295(2)	295(2)	295(2)
total no. of data collected	6832	4467	8130
no. of observed data	3090	2634	2655
no. of observed data (*I* > 2σ(*I*))	2677	2366	2506
*R*_1_, *wR*_2_ (*I* > 2σ(*I*))	0.0670, 0.1757	0.0380, 0.0773	0.0329, 0.0753
*R*_1_, *wR*_2_ (all data)	0.0759, 0.1868	0.0442, 0.0805	0.0353, 0.0766
no. of variable	169	162	162
GOF	1.038	0.990	0.988

*R*_F_ = ∑||*F*_o_ − *F*_c_||/∑|*F*_o_|; *R*_w_(*F*^2^) = [∑*w*|*F*_o_^2^ − *F*_c_^2^|^2^/∑*w*(*F*_o_^4^)]^1/2^.

## Supplementary Materials

The following are available online at http://www.mdpi.com/2073-4360/10/4/360/s1, Figure S1: A representation of a 2D structure of compound **1** connected by hydrogen bonding interactions. Figure S2. A 3D supramolecular network of compound **1** connected by hydrogen bonding interactions. Figure S3. A representation of the 3D network of **1** linked through hydrogen bonding interactions. Figure S4. These 2D layers of **3** are linked through hydrogen bonding interactions, leading to a 3D supramolecular network. Figure S5. Thermogravimetric analysis (TGA) curves of compounds: **1** (a), **2** (b), **3** (c). Figure S6. IR spectra of compounds: **1** (a), **2** (b), **3** (c). Figure S7. The powder X-ray diffraction (PXRD) patterns of **3** upon heating from room temperature up to 450 °C. Figure S8. PXRD patterns of **1** (under different temperatures), **2** and **3**. Figure S9. A view of local structure of **1** shows the Zn(II) ions between the neighboring carboxylate oxygen atoms (O_2_,O_4_) from the neighboring discrete complex with the separation distances of 4.2897 and 4.357 Å, respectively. Figure S10. A plausible mechanism of a structural transformation of **1** in the solid state, leading to the formation of a 1D helical chain of **2**. Figure S11. A presentation of structural transformation for **1**. This discrete complexes **1** were converted into **2** and **3** in the ratio of 97.6 to 2.4%. Table S1: Selected bond lengths (Å) and angles (°) for compound **1**. Table S2. Hydrogen bonding lengths (Å) and angles (°) for compound **1.** Table S3. Selected bond lengths (Å) and angles (°) for compound **2**. Table S4. Hydrogen bonding lengths (Å) and angles (°) for **2**. Table S5. Selected bond lengths (Å) and angles (°) for compound **3**. Table S6. Hydrogen bonding lengths (Å) and angles (°) for compound **3**.
