# Supplementary material for: Structural Transformations of Amino-Acid-Based Polymers: Syntheses and Structural Characterization
Source: Polymers (Basel). 2018 Mar 23;10(4):360. doi: 10.3390/polym10040360 (PMC6414969; doi:10.3390/polym10040360)
Supplement: Supplementary file 1 [file polymers-10-00360-s001.docx]

**Electronic Supplementary Information**

**Structural Transformations of Amino-Acid-Based Polymers: Syntheses and Structural Characterization**

Tien-Wen Tseng ^1,^*, Tzuoo-Tsair Luo ^1^, Hsiao-Shan Chiu ^2,3^, Chih-Chieh Wang ^3,^*,
Gene-Hsiang Lee ^4^, Hwo-Shuenn Sheu ^5^ and Kuang-Lieh Lu ^2,^*

^1^ Department of Chemical Engineering and Biotechnology, National Taipei University of Technology,
Taipei 106, Taiwan; luott@ntpc.edu.tw

^2^ Institute of Chemistry, Academia Sinica, Taipei 115, Taiwan; [anhhhh33@gmail.com](mailto:anhhhh33@gmail.com)

^3^ Department of Chemistry, Soochow University, Taipei 100, Taiwan

^4^ Department of Chemistry, National Taiwan University, Taipei 106, Taiwan; ghlee@ntu.edu.tw

^5^ National Synchrotron Radiation Research Center, Hsinchu 300, Taiwan; hsheu@nsrrc.org.tw

***** Correspondence: f10403tseng@gmail.com (T.-W.T.); ccwang@scu.edu.tw (C.-C.W);
kllu@gate.sinica.edu.tw (K.-L.L.)

1. Figures S1–S11

2. Tables S1–S6


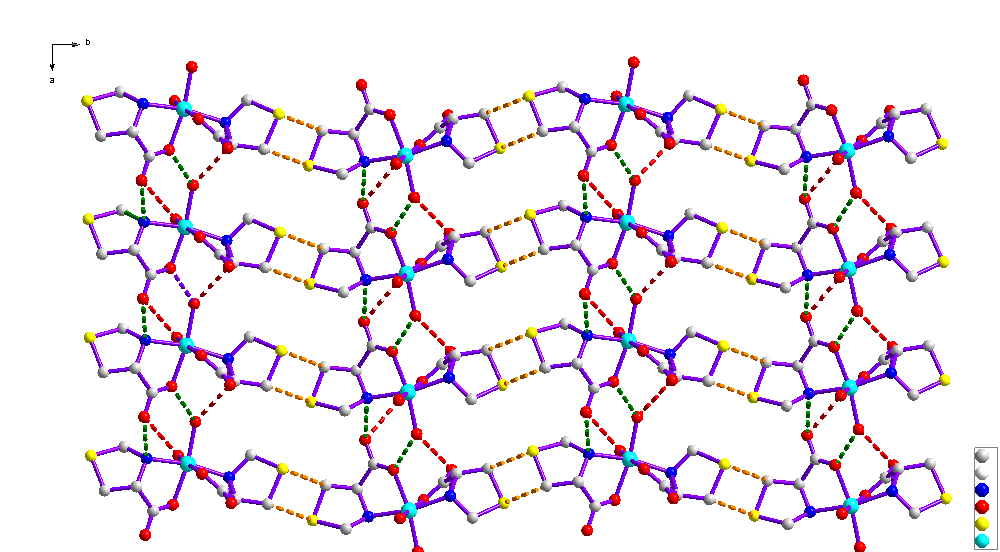


**Figure S1.** A representation of a 2D structure of compound **1** connected by hydrogen bonding interactions (yellow dash lines).


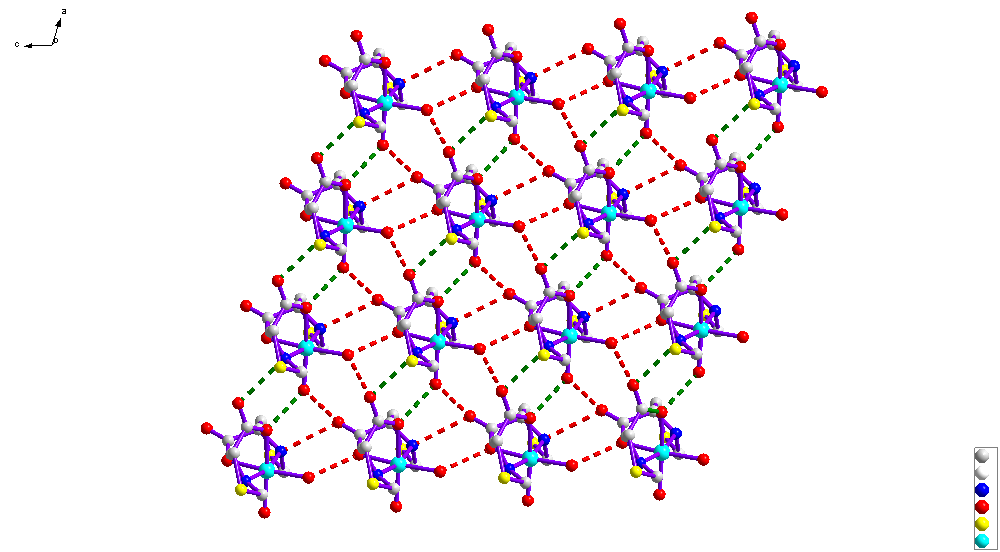


**Figure S2.** A 3D supramolecular network of compound **1** connected by hydrogen bonding interactions (green and red dash lines).

**Figure S3.** A representation of the 3D network of **1** linked through hydrogen bonding interactions (black dash lines).


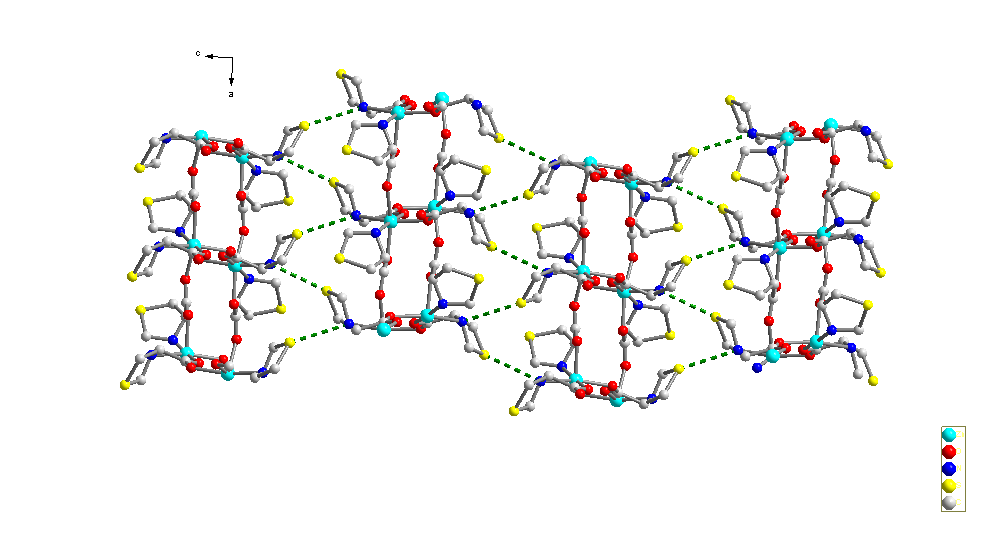


**Figure S4.** These 2D layers of **3** are linked through hydrogen bonding interactions (green dash lines), leading to a 3D supramolecular network.

(b)

(a)

(c)

**Figure S5.** Thermogravimetric analysis (TGA) curves of compounds: **1** (**a**), **2** (**b**), **3** (**c**).

(a)


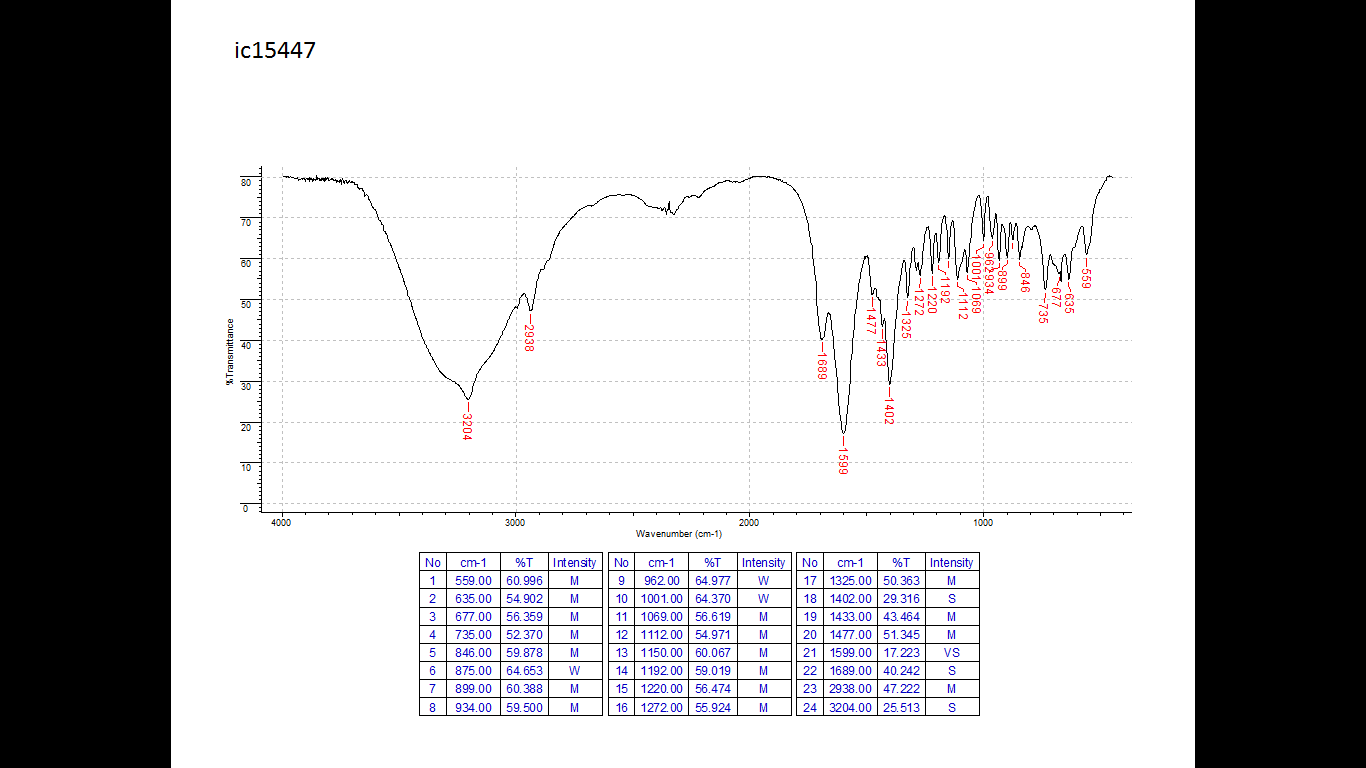


(b)

_
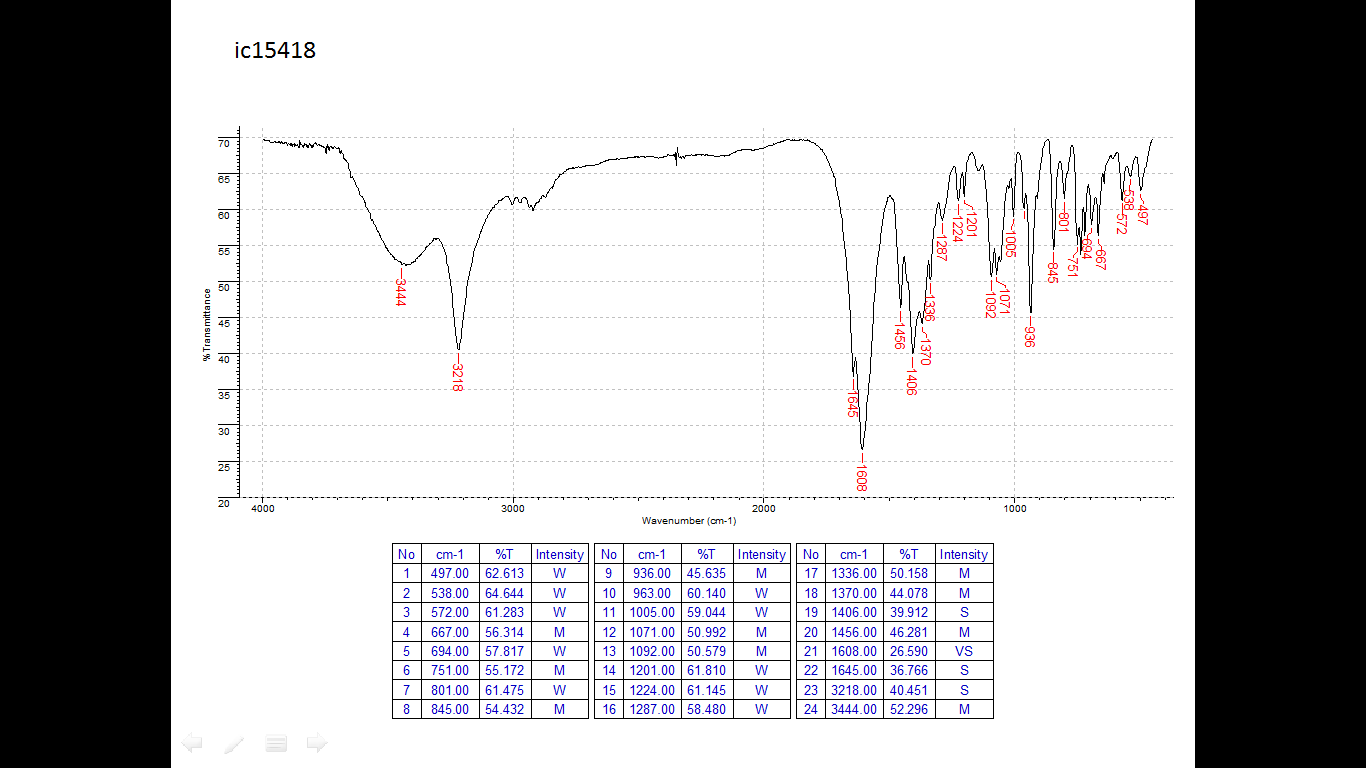
_


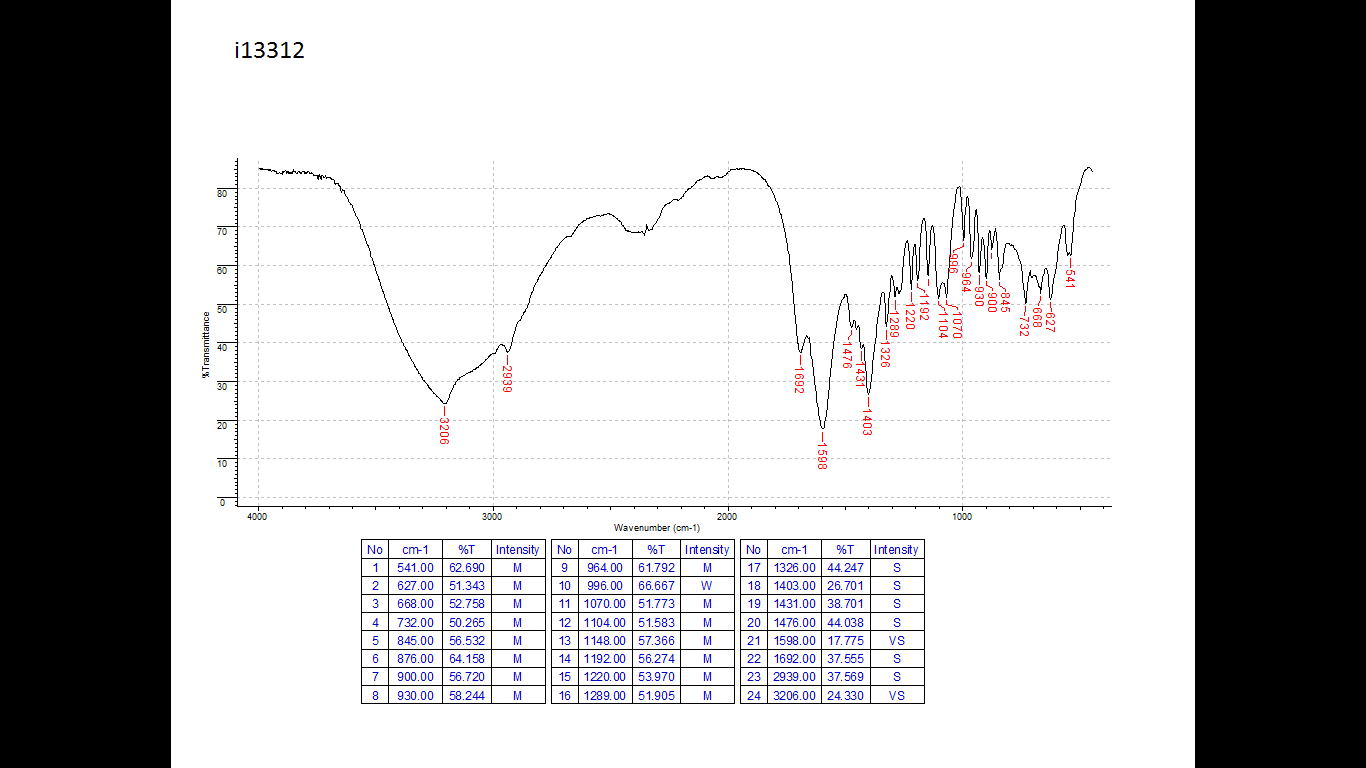


(c)

**Figure S6.** IR spectra of of compounds: **1** (**a**), **2** (**b**), **3** (**c**).

**Figure S7.** The powder X-ray diffraction (PXRD) patterns of **3** upon heating from room temperature up to 450 °C.

**[Zn(tpro)_2_(H_2_O)_2_]** **(1)**

**[Zn(tpro)_2_]*_n_*** **(2)**

**[Zn(tpro)_2_]*_n_*** **(3)**

**240** **°C**

**210** **°C**

**150** **°C**

**120** **°C**

**90** **°C**

**RT**

Two theta (degree)

**Figure S8.** PXRD patterns of **1** (under different temperatures), **2** and **3**.


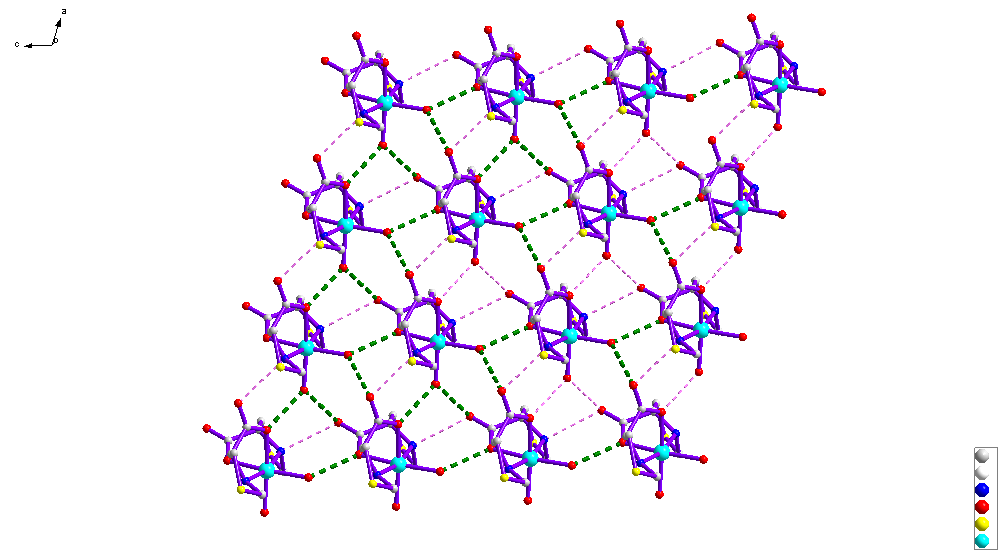


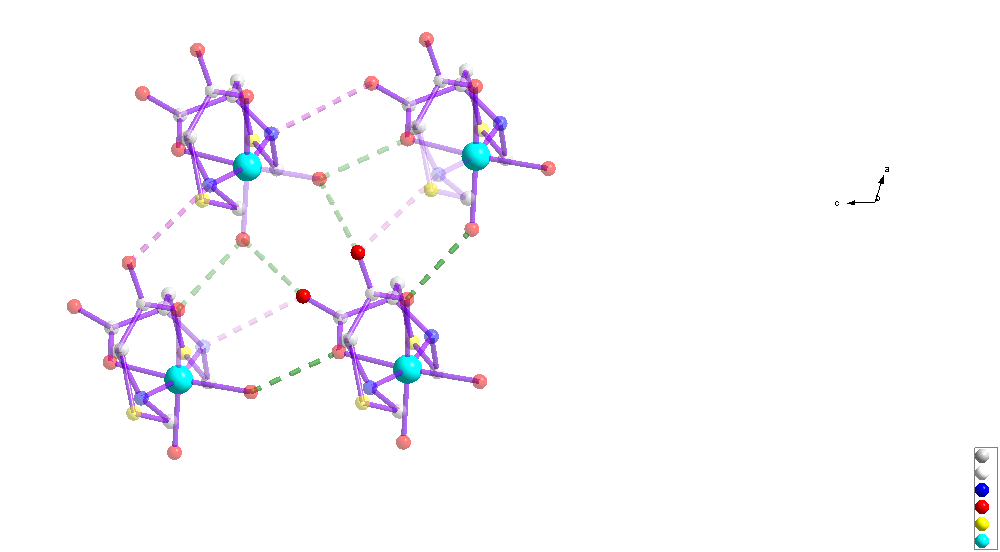


**4.297 Å**

**4.357 Å**

O4

**O2**

**Figure S9.** A view of local structure of **1** shows the Zn(II) ions between the neighboring carboxylate oxygen atoms (O2,O4) from the neighboring discrete complex with the separation distances of 4.2897 and 4.357 Å, respectively.


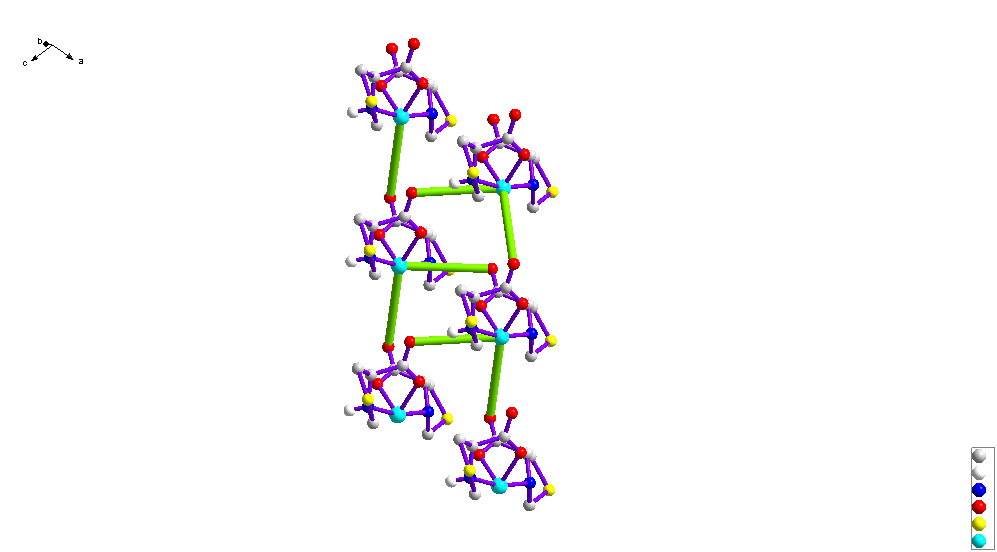

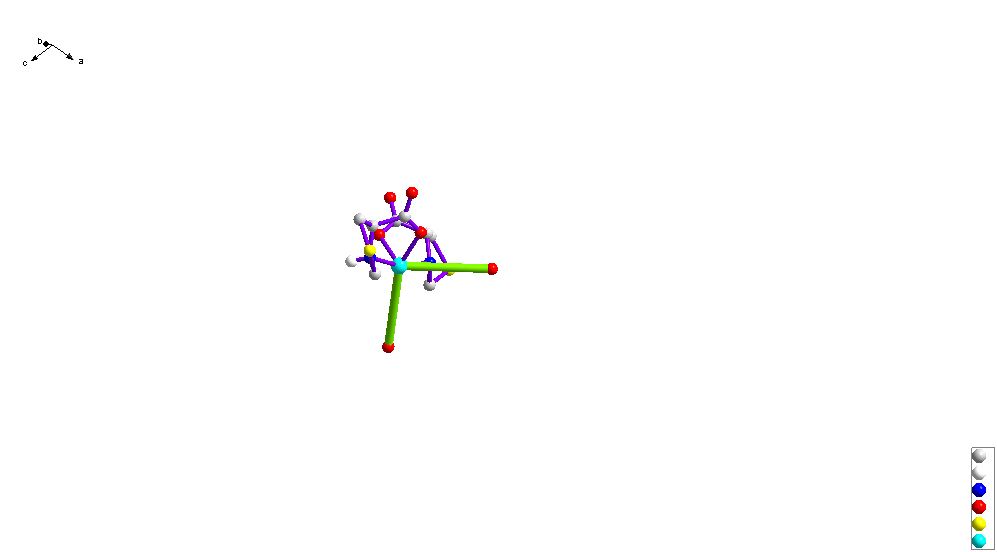

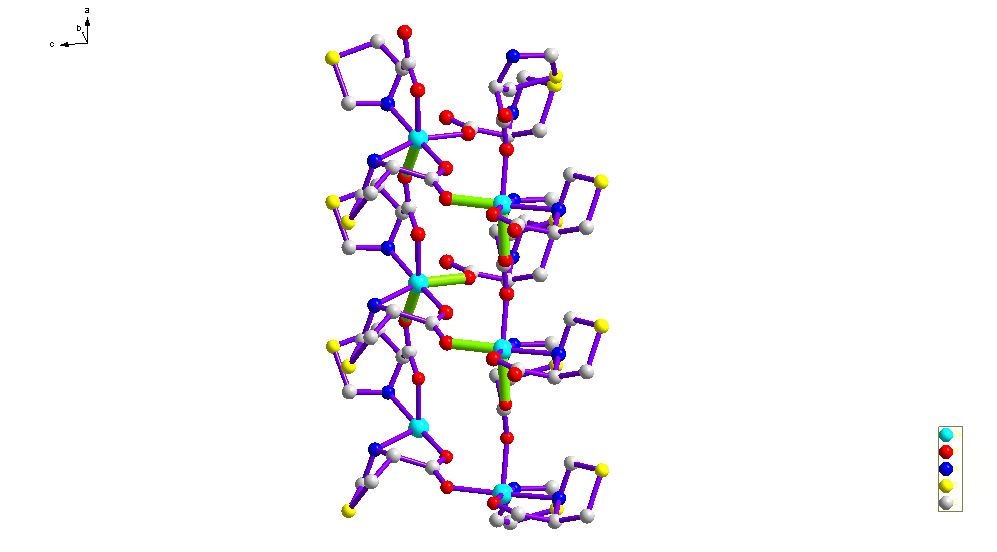

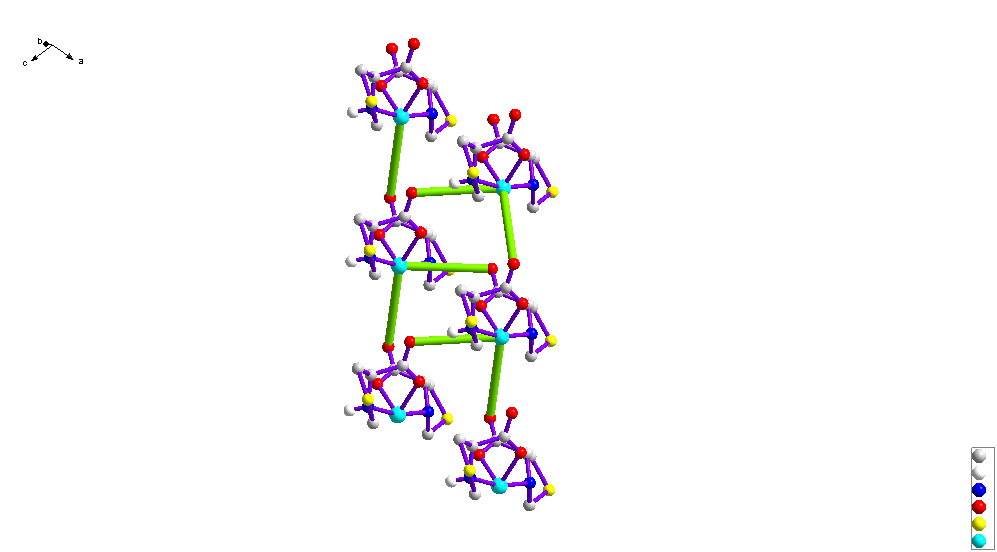

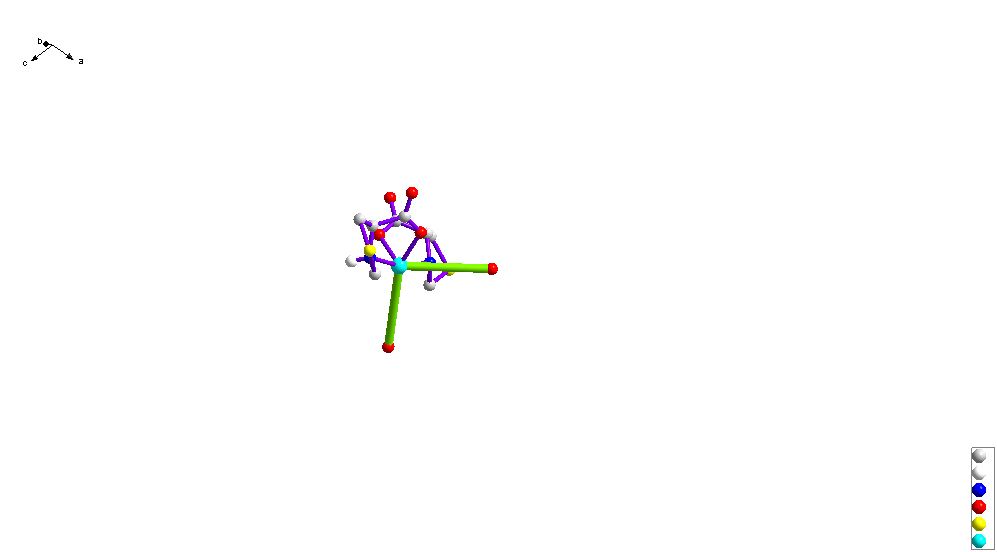


(a)


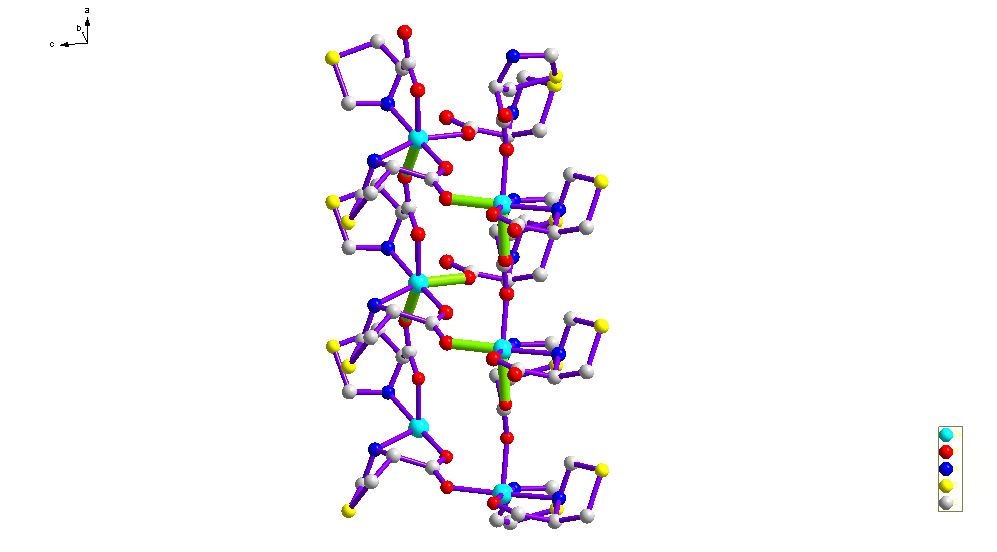


(b)

**Figure S10.** A plausible mechanism of a structural transformation of **1** in the solid state: (**a**) two coordinated water molecules were removed upon heating treatment, (**b**) the Zn(II) center created two uncoordinated sites. Additionally, the neighboring carboxylate motifs were also activated to be distorted and coordinated to the activated Zn(II) ions, leading to the formation of a 1D helical chain of **2**.


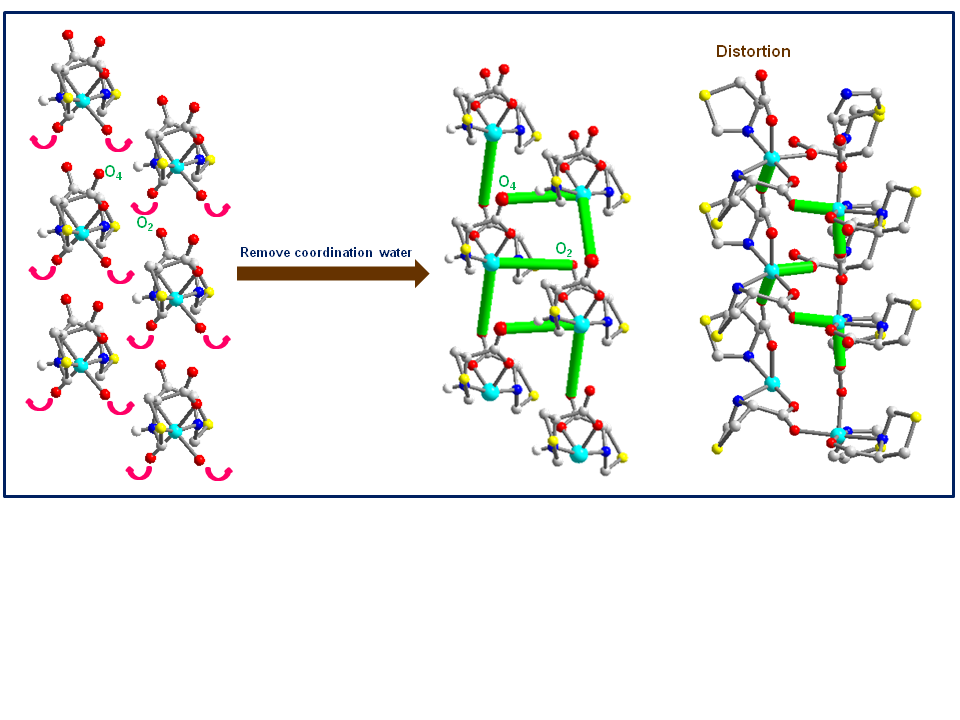


2.4 %

**Figure S11.** A presentation of structural transformation for **1**. This discrete complexes **1** were converted into **2** and **3** in the ratio of 97.6 to 2.4%, which was determined by the simulation of synchrotron PXRD data. It is probably attributed that the orientations of the carboxylate motifs on helical structure of **2** were difficult to be distorted.

**Table S1.** Selected bond lengths (°) and angles (°) for compound 1*^a^* .

| Zn−O(6) | 2.051(6) | Zn−O(5) | 2.060(6) |
| --- | --- | --- | --- |
| Zn−O(3) | 2.134(6) | Zn−N(2) | 2.142(1) |
| Zn−N(1) | 2.146(1) | Zn−O(1)_ii_ | 2.157(5) |
| O(6)−Zn−O(5) | 89.8(3) | O(6)−Zn−O(3) | 91.7(4) |
| O(5)−Zn−O(3) | 168.0(4) | O(6)−Zn−N(2) | 103.2(4) |
| O(5)−Zn−N(2) | 90.4(4) | O(3)−Zn−N(2) | 77.3(3) |
| O(6)−Zn−N(1) | 92.3(4) | O(5)−Zn−N(1) | 101.7(4) |
| O(3)−Zn−N(1) | 90.1(3) | N(2)−Zn−N(1) | 160.4(2) |
| O(6)−Zn−O(1) | 168.3(4) | O(5)−Zn−O(1) | 91.7(3) |
| O(3)−Zn−O(1) | 89.2(3) | N(2)−Zn−O(1) | 88.3(3) |
| N(1)−Zn−O(1) | 76.1(3) |  |  |

***^a^*** Symmetry codes used to generate equivalent atoms: ii = −1 + x, y, z.

**Table S2.** Hydrogen bonding lengths (Å) and angles (°) for compound **1***^a.^*

| D−H⋅⋅⋅A (Å) | D−H (Å) | H⋅⋅⋅A (Å) | D⋅⋅⋅A (Å) | ∠ D−H⋅⋅⋅A (°) |
| --- | --- | --- | --- | --- |
| N(1)−H(1)⋅⋅⋅O(2)_iii_ | 0.910(9) | 2.14 | 3.046(1) | 177.5 |
| N (2)−H(2)⋅⋅⋅O(4)_ii_ | 0.910(9) | 2.12 | 3.010(1) | 167.3 |
| O(5)−H(5A)⋅⋅⋅O(3)_ii_ | 0.844(1) | 1.81 | 2.636(1) | 166.4 |
| O(5)−H(5B)⋅⋅⋅O(2)_i_ | 0.859(9) | 1.86 | 2.695(1) | 175.1 |
| O (6)−H(6A)⋅⋅⋅O(1)_iii_ | 0.852(1) | 1.81 | 2.649(1) | 168.4 |
| O (6)−H(6B)⋅⋅⋅O (4)_i_ | 0.869(1) | 1.83 | 2.680(1) | 169.7 |
| C (1)−H(1B)⋅⋅⋅S(2) | 0.970(1) | 3.47 | 3.764(1) | 100.1 |
| C (5)−H(5C)⋅⋅⋅S(1) | 0.971(1) | 3.45 | 3.948(2) | 114.5 |

***^a^*** Symmetry codes used to generate equivalent atoms: i = −1 + x, y, −1 + z; ii = −1 + x, y, z; iii = x, y, −1 + z.

**Table S3.** Selected bond lengths (Å) and angles (°) for compound **2**.

| Zn−O(2)_i_ | 2.000(3) | Zn−O(3) | 2.047(3) |
| --- | --- | --- | --- |
| Zn−N(2) | 2.057(3) | Zn−N(1) | 2.075(3) |
| Zn−O(1) | 2.126(3) |  |  |
| O(2)_i_−Zn−O(3) | 95.50(1) | O(2)_i_−Zn−N(2) | 111.27(1) |
| O(3)−Zn−N(2) | 83.93(1) | O(2)_i_−Zn−N(1) | 130.99(1) |
| O(3)−Zn−N(1) | 93.34(2) | N(2)−Zn−N(1) | 117.57(2) |
| O(2)_i_−Zn−O(1) | 89.63(1) | O(3)−Zn−O(1) | 173.73 (1) |
| N(2)−Zn−O(1) | 97.58(1) | N(1)−Zn−O(1) | 80.55(1) |

***^a^*** Symmetry transformations used to generate equivalent atoms: i = −x+1, y−1/2, −z.

**Table S4.** Hydrogen bonding lengths (Å) and angles (°) for **2**.

| D−H⋅⋅⋅A (Å) | D−H (Å) | H⋅⋅⋅A (Å) | D⋅⋅⋅A (Å) | ∠ D−H⋅⋅⋅A (°) |
| --- | --- | --- | --- | --- |
| N(1)−H(1)⋅⋅⋅O(4)_i_ | 0.682(4) | 2.27 | 2.862(5) | 145.7 |
| N(2)−H(2)⋅⋅⋅O(3)_ii_ | 0.792(4) | 2.50 | 3.170(5) | 142.3 |
| N(2)−H(2)⋅⋅⋅O(4)_i_ | 0.792(4) | 2.59 | 3.097(6) | 123.2 |
| C(6)−H(6A)⋅⋅⋅S(2)_iii_ | 0.980(5) | 2.81 | 3.518(6) | 130.1 |

***^a^*** Symmetry codes used to generate equivalent atoms: i = 1 − x, 1/2 + y, 1 − z; ii = x, 1 + y, z; iii = −x, −1/2 + y, 1 − z.

**Table S5.** Selected bond lengths (Å) and angles (°) for compound **3**.

| Zn(1)−O(1) | 2.033(2) | Zn(1)−O(4)_i_ | 2.079(3) |
| --- | --- | --- | --- |
| Zn(1)−O(2)_ii_ | 2.100(2) | Zn(1)−O(3) | 2.130(3) |
| Zn(1)−N(2) | 2.151(2) | Zn(1)−N(1) | 2.295(2) |
| O(1)−Zn(1)−O(3) | 96.22(1) | O(2)_ii_−Zn(1)−O(1) | 85.18(1) |
| O(4)_i_−Zn(1)−O(2)_ii_ | 87.52(1) | O(2)_ii_−Zn(1)−O(3) | 99.22(1) |
| O(4)_i_−Zn(1)−O(3) | 160.32(1) | O(1)−Zn(1)−O(4)_i_ | 102.79(1) |
| O(1)−Zn(1)−N(2) | 173.66(1) | O(4)_i_−Zn(1)−N(2) | 83.09(1) |
| O(2)_ii_−Zn(1)−N(2) | 97.56(1) | O(1)−Zn(1)−N(1) | 75.96(1) |
| O(3)−Zn(1)−N(2) | 77.72(1) | O(3)−Zn(1)−N(1) | 88.26(1) |
| O(2)_ii_−Zn(1)−N(1) | 160.40(1) | O(4)_i_−Zn(1)−N(1) | 91.42(1) |
| N(2)−Zn(1)−N(1) | 101.74(1) |  |  |

***^a^*** Symmetry transformations used to generate equivalent atoms: i = x+1, y, z; ii = −x+2, y+1/2, −z+1/2.

**Table S6.** Hydrogen bonding lengths (Å) and angles (°) for compound **3.**

| D−H⋅⋅⋅A (Å) | D−H (Å) | H⋅⋅⋅A (Å) | D⋅⋅⋅A (Å) | ∠ D−H⋅⋅⋅A (°) |
| --- | --- | --- | --- | --- |
| N(1)−H(1B)⋅⋅⋅S(1)_ii_ | 0.872(4) | 2.64 | 3.418(2) | 145.7 |
|  |  |  |  |  |

***^a^*** Symmetry codes used to generate equivalent atoms: i = 1−x, 1/2+y, 1−z.

© 2018 by the authors. Submitted for possible open access publication under the
terms and conditions of the Creative Commons Attribution (CC BY) license (http://creativecommons.org/licenses/by/4.0/).
